# Cryo-EM structures of τ filaments from human brain

**DOI:** 10.1042/EBC20210025

**Published:** 2021-12-22

**Authors:** Michel Goedert

**Affiliations:** MRC Laboratory of Molecular Biology, Francis Crick Avenue, Cambridge CB2 0QH, U.K.

**Keywords:** cryo-electron microscopy, molecular conformation, tau proteins

## Abstract

Electron cryo-microscopy (cryo-EM) has made it possible to determine near-atomic structures of τ filaments from human brain. Previous work had shown that the cores of paired helical and straight filaments of Alzheimer's disease are made of two identical, but differently arranged C-shaped protofilaments. In recent years, cryo-EM has shown that the Alzheimer τ fold is 79 amino acids long. Five of the eight β-strands give rise to two antiparallel β-sheets, with the other three forming a β-helix. High-affinity binding sites of positron emission tomography ligand APN-1607 (PM-PBB3) are in the β-helix region. The Alzheimer fold contrasts with the 94 amino acid-long Pick fold, which is J-shaped and comprises nine β-strands that give rise to four antiparallel β-sheets, in the absence of a β-helix. Chronic traumatic encephalopathy τ fold is similar to the Alzheimer fold, but differs in the β-helix region, which is larger and contains a non-proteinaceous density that is probably hydrophobic. These folds are mostly two-layered. By contrast, the 107 amino acid τ fold of the 4R tauopathy corticobasal degeneration is four-layered and comprises 11 β-strands. It contains an internal, probably hydrophilic, density that is surrounded by τ. The τ folds described here share the presence of microtubule-binding repeats 3 and 4, as well as 10–13 amino acids after repeat 4.

## Introduction

Filamentous deposits of known composition in brain cells define many human neurodegenerative diseases, including Alzheimer's and Parkinson's. The brain cells in which deposits form degenerate, resulting in dementias and movement disorders, depending on which parts of the central nervous system are affected. Most cases of disease are idiopathic, but some are inherited in a dominant manner (Huntington's disease and other trinucleotide disorders are always inherited, whereas chronic traumatic encephalopathy (CTE) is environmentally induced).

A central role for filament formation was identified when inherited cases of disease were shown to be caused by mutations in the genes that encode the major filament components, be they τ, α-synuclein, Aβ, prion protein, TDP-43 or FUS. Mutations in *MAPT*, the τ gene, give rise to familial forms of frontotemporal dementia [1]. Abnormal assembly and deposition of the mutant proteins may therefore represent a gain of toxic function, which underlies the development of disease. By extrapolation, it follows that a similar gain of toxic function resulting from the ordered assembly into filaments may also underlie idiopathic forms of disease. Abnormal protein deposition appears to follow spatiotemporal spreading, suggesting that pathology may propagate through seeded aggregation, similar to what happens in prion diseases [1].

Here, I provide an overview of the structures of several conformers of assembled τ from human brain that have been determined by electron cryo-microscopy (cryo-EM) and their possible significance for diagnosis and pathogenesis [2–7].

## τ protein and its isoforms

**Figure 1:**
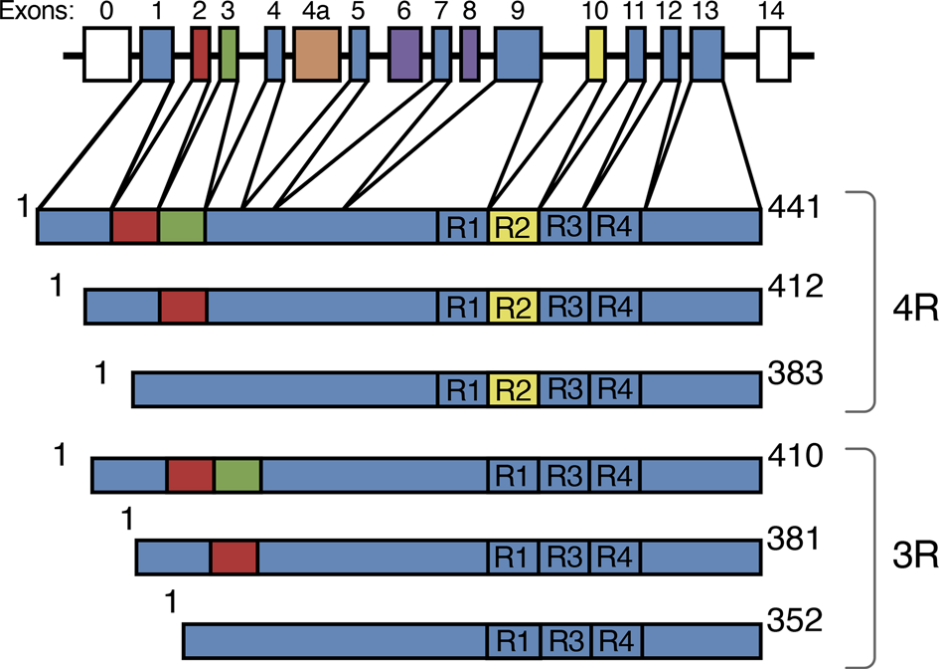
Human brain τ isoforms *MAPT* and the six τ isoforms expressed in adult human brain. *MAPT* consists of 14 exons (E). Alternative mRNA splicing of E2 (red), E3 (green) and E10 (yellow) gives rise to six τ isoforms (352–441 amino acids). Constitutively spliced exons (E1, E4, E5, E7, E9, E11, E12, E13) are shown in dark blue. E6 and E8 (violet) are not transcribed in human brain. E4a (orange) is only expressed in the peripheral nervous system. The repeats (R1–R4) are shown, with three isoforms having four repeats (4R) and three isoforms having three repeats (3R).

τ is an intrinsically disordered protein, with many potential interaction partners. It can be divided into an N-terminal domain, a proline-rich region, the repeat domain and a C-terminal region (Figure 1) [1]. The N-terminal domain projects from the microtubule surface and is believed to interact with components of the plasma membrane. It contains a primate-specific sequence between residues 19 and 29 of human τ (GLGDRKDQGGY). The PXXP motifs in the proline-rich region are recognised by SH3 domain-containing proteins of the Src family of non-receptor tyrosine kinases, such as Fyn.

The repeat region and some adjacent sequences mediate interactions between τ and microtubules. By cryo-EM, each τ repeat binds to the outer microtubule surface and adopts an extended structure along protofilaments, interacting with α- and β-tubulins [8]. Single-molecule tracking has revealed a kiss-and-hop mechanism, with a dwell time of τ on individual microtubules of approximately 40 milliseconds. Isoform differences did not influence this interaction. Despite these rapid dynamics, τ promoted microtubule assembly. It remains to be seen if microtubules were stabilised. τ is most abundant in the labile domain of microtubules, which has led to the suggestion that it may not stabilise microtubules, but enable them to have long labile domains [9]. Less is known about the function of the C-terminal region, which may inhibit assembly into filaments.

Despite the absence of a typical low-complexity domain, τ can undergo liquid–liquid phase separation through electrostatic and hydrophobic interactions in conjunction with amyloid aggregation, at least *in vitro* [10]. Although liquid–liquid phase separation and amyloid aggregation are independent processes, they may be able to influence each other.

Six τ isoforms that range from 352 to 441 amino acids in length are expressed by alternative mRNA splicing from the *MAPT* gene in adult human brain (Figure 1) [11]. They differ by the presence or absence of inserts of 29 and 58 amino acids (encoded by exons 2 and 3, with exon 3 being only transcribed together with exon 2) in the N-terminal half, and the inclusion, or not, of the 31 amino acid repeat, encoded by exon 10, in the C-terminal half. Inclusion of exon 10 results in the production of three isoforms with four repeats (4R) and its exclusion in a further three isoforms with three repeats (3R). The repeats comprise residues 244–368, in the numbering of the 441 amino acid isoform. In adult human brain, similar levels of 3R and 4R τ are expressed. It came as a surprise to find that a correct 3R/4R τ isoform ratio is essential for preventing neurodegeneration and dementia. Thus, the relative overproduction of either 3R or 4R τ, as a result of coding region or intronic mutations in *MAPT*, gives rise to dominantly inherited frontotemporal dementias. 2N isoforms are underrepresented relative to those that include exon 2 or exclude both exons 2 and 3; 2N, 1N and 0N isoforms make up 9, 54 and 37%, respectively, of total brain τ. Big τ, which carries an additional large exon in the N-terminal half, is only expressed in the peripheral nervous system [1].

τ isoform expression is not conserved between species. Thus, in adult mouse brain, only 4R τ isoforms are present, whereas adult chicken brain expresses 3R, 4R and 5R τ. However, the presence of a single hyperphosphorylated 3R τ isoform that lacks N-terminal inserts is characteristic of developing vertebrates. In mice, the switch from 3R to 4R τ occurs between postnatal days 9 and 18, with τ phosphorylation decreasing over time. Isoform switching and phosphorylation are regulated differently [12].

## τ assemblies

In 1907, using Bielschowsky silver, Alzheimer described the neurofibrillary tangles [13] that are characteristic of the disease Emil Kraepelin subsequently named after him. In 1911, Alzheimer also described the light microscopic appearance of what we now know as the Pick body [14]. The principal filamentous components of the tangles were first visualised with the electron microscope by Kidd in 1963 [15]. He described them as consisting of two strands that are helically wound around each other and aptly named them ‘paired helical filaments’ (PHFs) [15]. Tangles consist predominantly PHFs, but they also contain morphologically distinct straight filaments (SFs) as a minor component. Between 1985 and 1991, PHFs and SFs were shown to be made of τ protein [16–19].

Around this time, τ was also shown to be the major component of the filamentous inclusions of Pick's disease and progressive supranuclear palsy (PSP) [20]. The same is also true of other conditions, such as primary age-related tauopathy (PART), corticobasal degeneration (CBD), globular glial tauopathy (GGT) and argyrophilic grain disease (AGD) [1]. Negative-stain immunoelectron microscopy (immuno-EM) showed that antibodies specific for the N- and C-termini of τ decorate filaments [21]. This was not the case of antibodies directed against R3 and R4, because their epitopes are occluded. Together with biochemical studies, this work established that τ filaments consist of a structured core made of at least some of the repeats and a less structured fuzzy coat made of the bulk of τ [22,23]. Electron diffraction of individual τ filaments extracted from Alzheimer's disease (AD) brain established their amyloid nature [24].

τ filaments are post-translationally modified [1], with abnormal phosphorylation being the most widely studied. But other modifications are also present, including acetylation, methylation, glycation, isomerisation, O-GlcNAcylation, nitration, sumoylation, ubiquitination and truncation. It remains to be seen if abnormal phosphorylation and other modifications are necessary and/or sufficient for the assembly of τ into filaments. Alternatively, a change in conformation may lead to τ hyperphosphorylation and other modifications. Acetylation of lysine residues in τ has come to the fore in recent years. It reduces charge, which may play a role in filament assembly. Site-specific acetylation of K280 has been shown to enhance τ aggregation, while reducing microtubule assembly [25]. Eleven lysines are present in Alzheimer and CTE τ folds, and 16 lysines in Pick and CBD folds.

Since τ is very soluble, it is not surprising that unmodified, full-length recombinant protein requires cofactors, such as sulphated glycosaminoglycans, free fatty acids and nucleic acids, to assemble into filaments [26–29]. However, the cryo-EM structures of filaments assembled by adding heparin to recombinant τ are different from those of filaments extracted from human brain [30]. It has been known for close to 30 years that the repeat region of τ can assemble into filaments in the absence of cofactors [31–33]. It is therefore conceivable that the positively charged region upstream of the repeats inhibits the assembly of full-length τ into filaments. Phosphorylation of amino acids in this region [34] may be necessary for filament assembly. However, additional cellular factors may also be required and, depending on their nature, different τ folds may form.

In AD, CTE, tangle-only dementia and other tauopathies, all six brain τ isoforms (3R+4R) are present in disease filaments. Pick bodies are made only of 3R τ. In CBD, AGD, PSP, GGT and other diseases, 4R τ isoforms make up the filaments. Morphologies and structures of filaments vary, even when they are made of the same τ isoforms. Biochemically, the banding pattern of C-terminal fragments of τ differs between PSP and CBD, with GGT being like PSP and AGD like CBD [35–37].

## Conformers of assembled τ

Distinct conformers of assembled τ exist, reminiscent of prion strains. This may explain at least some of the variety of human tauopathies. Inclusions formed after intracerebral inoculation of brain homogenates from cases of AD, tangle-only dementia, PiD, AGD, PSP and CBD into the mouse line transgenic for 2N4R human τ [38,39]. As judged by their light microscopic appearance and presence in nerve cells and glial cells, τ assemblies reminiscent of human disorders were observed following the injection of brain homogenates from patients with AGD, CBD and PSP. A smaller number of similar inclusions also formed after the intracerebral injection of brain homogenates from human tauopathies into wildtype mice.

Conformationally distinct τ assemblies made of four τ repeats formed in transfected non-neuronal cells following exposure to seeds from human tauopathies [40]. Inoculation of these seeded assemblies into the hippocampus of young P301S τ transgenic mice induced the formation of assemblies that were stable through serial transmission. When cells expressing four τ repeats were seeded with homogenates from these brains, inclusions formed that were identical with those present initially.

Taking everything together, these findings suggest that distinct conformers of assembled τ underlie different human tauopathies.

## Structures of τ filaments from AD

AD, the most common neurodegenerative disease, is an Aβ and τ proteinopathy. τ inclusions are made of all six brain isoforms and are largely neuronal [21]. In 1991, electron microscopy and image reconstruction were used to show that PHFs and SFs are made of identical C-shaped protofilaments that are linked in different ways [41], but individual amino acids could not be visualised. This had to wait until 2017, when cryo-EM was used to determine the near-atomic structures of τ filaments from the brain of a patient with AD [2].

**Figure 2:**
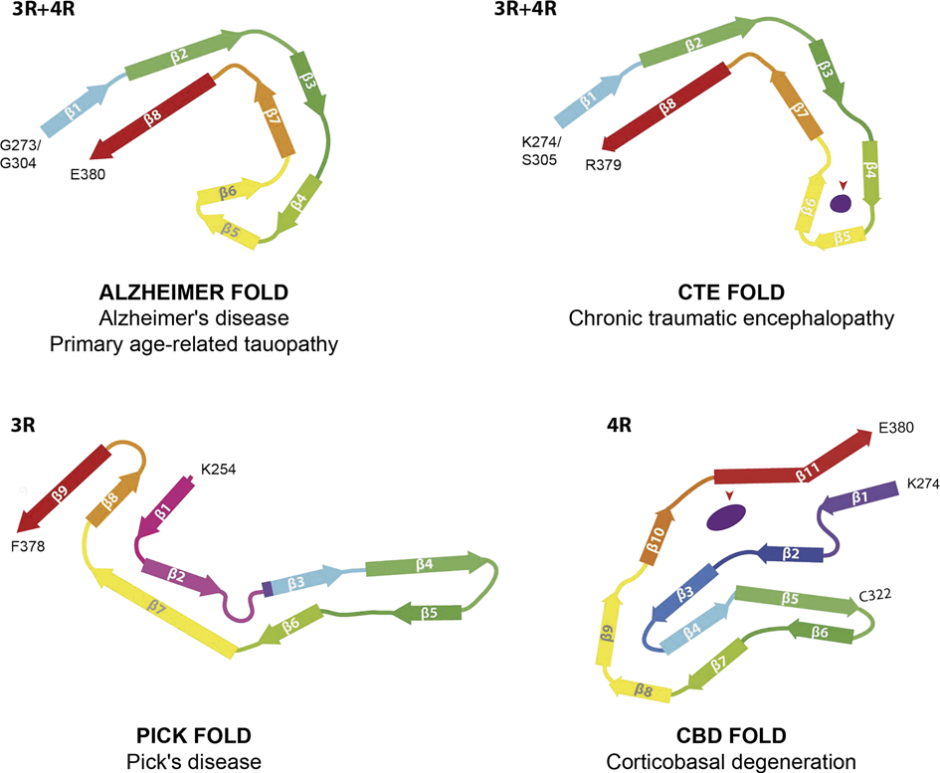
Structures of τ protofilaments from human brain Protofilaments shown through the XY-plane, perpendicular to the helical axis. β-strands correspond to coloured, arrowed bars. Red arrowheads point to internal densities. Fuzzy densities on the outside of protofilaments are not shown.

The C-shaped τ fold comprises 79 amino acids; it starts at G273 for 3R and G304 for 4R τ isoforms and ends at E380 (Figure 2) [2,4,7]. These sequences correspond to the carboxy-terminal two amino acids of R1 and R2, the whole of R3 and R4, and 12 amino acids after R4. Even though the core of τ filaments may extend by a few more amino acids, immuno-EM has shown that an antibody raised against a peptide corresponding to residues 275–291 of 4R τ decorates PHFs and SFs, indicating that this region forms part of the fuzzy coat. The same is true of residue E391 [2].

The cores comprise eight β-strands (β1–β8) in a two-layered, C-shaped arrangement. A heterotypic interface forms between β1–2 and β8. β-1 consists of ^306^VQIVYK^311^, which forms a packing interface with amino acids 373–378 from β8. Strands β2 and β8 pack against each other through a polar-zipper motif, with a second antiparallel β-sheet between β3 and β7. The two sides meet through a β-helix that consists of β4–β6 from R4.

**Figure 3:**
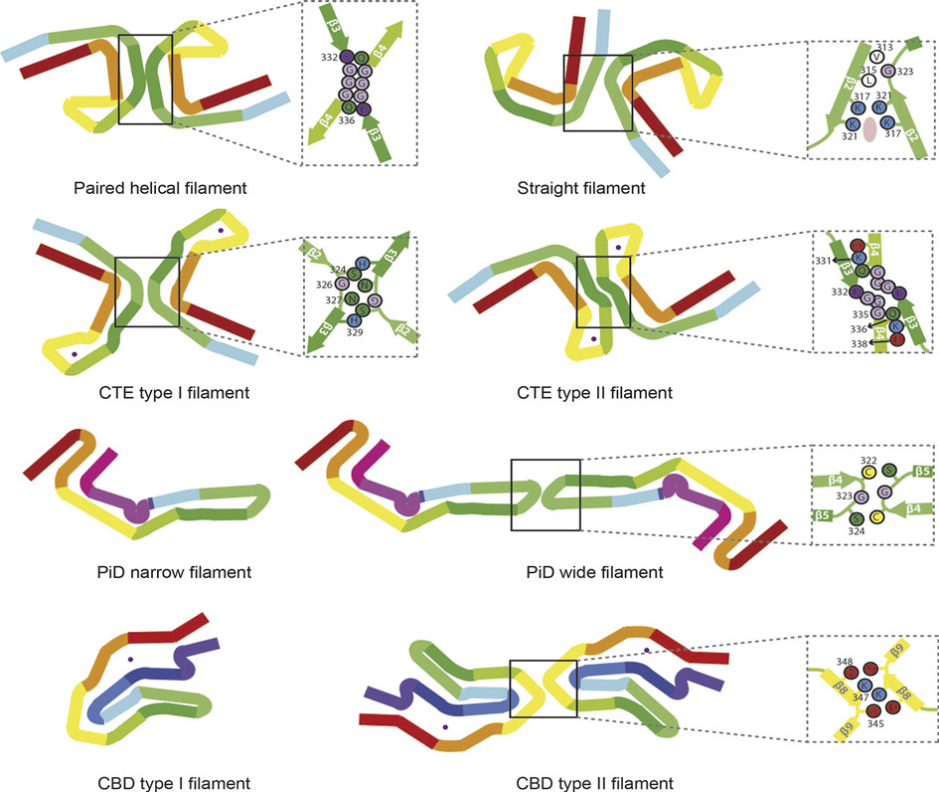
Two filament types are characteristic of each disease One or two identical protofilaments are present. Two protofilaments are always seen in AD PHFs and SFs, as well as in CTE type I and CTE type II filaments. Both filament types are ultrastructural polymorphs. In Pick's disease (PiD) and CBD, single or double protofilaments are present. Zoomed-in schematics show amino acids involved in protofilament interfaces. Pink oval represents the additional density in the straight filament of Alzheimer's disease (AD SF).

PHFs and SFs are made of the same protofilament pairs. They are ultrastructural polymorphs, because they differ in lateral contacts (Figure 3). In PHFs, the interface is stabilised by hydrogen bonding interactions between K331 from one protofilament and Q336 and E338 from the other. In SFs, protofilaments pack asymmetrically and are stabilised by an additional, possibly anionic, density between the side chains of K317 and K321 from both protofilaments.

The first structures were from frontal cortex of a patient with sporadic AD [2]. We have since determined identical structures of τ filaments from the frontal cortex of three additional patients with AD [two sporadic and one familial (V717F mutation in the amyloid precursor protein gene)] and from occipital cortex of a patient with posterior cortical atrophy (PCA), a subtype of AD [4,7]. The Alzheimer τ fold was also found in some cases of prion protein amyloidosis [42], indicating that it can coexist with extracellular amyloid deposits. Moreover, the Alzheimer τ fold was present in the limbic region from three cases of PART [7], indicating that it can form in the absence of extracellular amyloid deposits. Aβ has been shown to promote, but not initiate, the Alzheimer τ fold [43,44]. It remains to be seen if the Alzheimer τ fold forms constitutively in human brain. τ inclusions have been detected in the brains of asymptomatic children and young adults [45].

## Structures of τ filaments from AD with positron emission tomography ligand APN-1607 (PM-PBB3)

**Figure 4:**
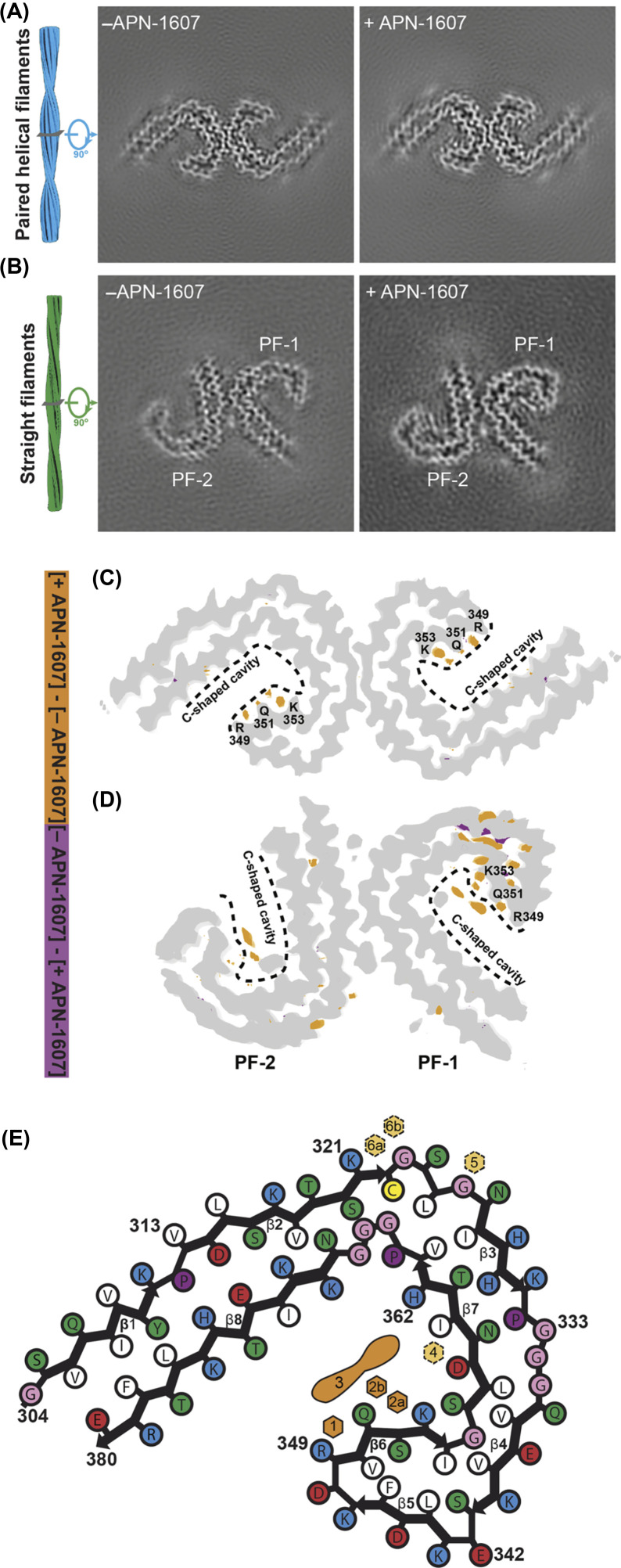
Cryo-EM maps of PHFs and SFs from AD with APN-1607 (**A**,**B**) Cryo-EM maps of τ filaments without (−APN-1607) and with (+APN-1607) PET ligand. (**C**,**D**) Overlay of positive (orange) and negative (purple) difference maps and −APN-1607 maps (grey). SF protofilaments 1 and 2 are labelled as PF-1 and PF-2. Amino acids R349, Q351 and K353 are indicated, with the C-shaped cavities outlined by stippled lines. The thresholds in the difference maps were 11 standard deviations for PHFs and 8 standard deviations for SFs. (**E**), Alzheimer τ protofilament core with APN-1607 binding sites. Major binding sites 1, 2a, 2b and 3 are shown in orange; minor binding sites 4, 5, 6a and 6b are indicated in yellow.

APN-1607 (PM-PBB3) is a positron emission tomography (PET) ligand for AD and some other tauopathies [46]. Since it is not known where in the τ folds PET ligands bind, we used cryo-EM to determine the binding sites of APN-1607 in the Alzheimer fold [7]. We identified two major sites in the β-helix region of PHFs and SFs (binding sites 1, 2a and 2b) and a third major site in the C-shaped cavity of SFs (binding site 3) (Figure 4). Binding site 1 was in the groove between the side chains of R349 and Q351 from β6. Binding sites 2a and 2b in the groove between the side chains of Q351 and K353. Binding site 3 was in the C-shaped cavity of SFs.

Knowledge of the binding modes of APN-1607 to τ filaments may lead to the development of new ligands with increased specificity and binding activity. More generally, these findings have established that cryo-EM can be used to identify the binding sites of small molecules in amyloid filaments. It remains to be seen if APN-1607 binds to τ filaments other than those from AD and where in τ filaments different PET ligands bind.

## Structures of τ filaments from CTE

CTE is associated with repetitive head impacts and exposure to blast waves. τ inclusions are neuronal and glial. Nerve cell inclusions, which are in the majority, are made of all six brain τ isoforms [47]. Astrocytic inclusions have been reported to consist of 4R τ.

By negative-stain EM, we observed a major helical filament type in two ex-boxers and a former American football player, who had died with a neuropathologically confirmed diagnosis of stage IV CTE. The remaining filaments resembled Alzheimer PHFs. Filament structures were determined to a resolution of 2.3 Å [5]. They comprise residues K274-R379 of 3R τ and S305-R379 of 4R τ which form the ordered core of two identical C-shaped protofilaments (Figures 2 and 3). The secondary structures of Alzheimer and CTE folds comprise eight β-strands. The tip of the C is formed by β4–β6, where the packing coincides with an opening in the CTE fold. The resulting cavity is filled with a density that is not connected to τ, consistent with the presence of additional molecules of unknown identity. The nature of the cavity suggests that these molecules are hydrophobic.

In CTE type I filaments (90% by negative staining), two identical protofilaments pack in a staggered manner through an antiparallel steric zipper formed by residues ^324^SLGNIH^329^ (Figure 3). In CTE type II filaments (7% by negative staining, 3% were PHFs), the protofilament interface is also staggered and comprises the same residues as in PHFs, but a kinked conformation reduces the number of hydrogen bonds across the interface. These findings provide a unifying criterion for stage IV CTE and confirm that dementia pugilistica and CTE are the same disease. They also establish that AD and CTE are distinct τ proteinopathies.

## Structures of τ filaments from Pick's disease

PiD is a type of frontotemporal dementia with mostly neuronal inclusions that are made of 3R τ [48]. We observed narrow (>90%) and wide (<10%) Pick filaments (NPFs and WPFs) by negative staining [3]. By cryo-EM, we determined a 3.2 Å resolution map of the core of NPFs from frontotemporal cortex of a case of sporadic PiD. A single protofilament extends from K254 to F378 of 3R τ, comprising 94 amino acids (Figure 2). It consists of the C-terminal 21 amino acids of R1, the whole of R3 and R4, and 10 amino acids after R4. Nine β-strands adopt a J shape and are arranged into four cross-β packing stacks, which are connected by turns and arcs; β1, β8 and β9 form a three-layered motif, with the rest of the J containing two layers. In WPFs, two protofilaments pack symmetrically against each other through Van der Waals interactions at the tip of the J (Figure 3).

To investigate the generality of the Pick fold, we used immuno-EM of τ filaments from frontotemporal cortex of eight additional patients with sporadic PiD. Most filaments were NPFs, with a minority of WPFs; they were not decorated by repeat-specific antibodies. This showed that R1, R3 and R4 epitopes were inaccessible, indicating that they form part of the filament core. In keeping with the absence of τ phosphorylation at S262 and/or S356 in PiD [49], the tight turn at G261 of NPFs prevents phosphorylation of S262.

## Structures of τ filaments from CBD

CBD is characterised by motor and cognitive disturbances, with 4R τ inclusions forming in nerve cells and glial cells [50]. Astrocytic plaques are pathognomonic [51]. We observed narrow (Type I) and wide (Type II) filaments by negative staining, with ratios of 1:1 to 1:3 [6]. The structures of Type I and Type II filaments were determined to resolutions of 3.2 and 3.0 Å. Type I filaments are composed of a protofilament that adopts a compact four-layered fold (Figure 2). It contains a density that is surrounded by τ protein. Unlike what is observed in CTE, this internal density is located in a positively charged environment. Type II filaments consist of pairs of Type I protofilaments, related by C2 symmetry (Figure 3). The protofilament interface is formed by antiparallel stacking of ^343^KLDFKDR^349^. The CBD fold extends from K274-E380 of 4R τ, comprising 107 amino acids. The core of Type I filaments consists of the last residue of R1, the whole of R2–R4 and 12 amino acids after R4. They comprise 11 β-strands. The central four layers are formed by β7, β4, β3 and β10. On one side, the residues that form β7–β9 in the CBD fold are the same as β4–β6 in Alzheimer and CTE folds. On the other side, the hairpin structure formed by β5 and β6 is similar to the conformation between β4 and β5 of the Pick fold. Fuzzy densities are present on the outside of filaments, often in close proximity of the side chains of lysine residues and solvent-exposed hydrophobic amino acid patches. It has been suggested [52] that some of these densities may correspond to ubiquitin and that differential ubiquitination of lysine residues could explain differences between the τ folds of AD and CBD.

## Conclusion

In tauopathies, monomeric, soluble intracellular τ assembles into insoluble filaments. During the past 12 years, multiple lines of evidence have shown that assembled τ behaves like a prion. It is clear that τ seeds can template the assembly of monomeric protein, but it remains to be established what role the prion-like spreading of assembled τ plays in human brain.

Over the past 5 years, structure determination of τ filaments from human brain by cryo-EM has provided evidence for the existence of multiple conformers, reminiscent of prion strains. It is possible that differences between τ isoforms, post-translational modifications and associated, non-proteinaceous molecules, account for this diversity. Even though most phosphorylation sites in τ are located outside the core of PHFs and SFs, other modifications of the core, such as acetylation and ubiquitination, may also play a role. Differences in structure are between diseases, not between individuals with a given disease. It is not known if rare, structurally different filaments exist that might have been missed. In CTE, PHFs, which accounted for 3% of filaments, could be distinguished from Type I and Type II τ filaments by cryo-EM [5].

The cellular milieu may also be crucial for the formation of disease seeds. It is now possible to use cryo-EM to compare the structures of disease seeds and seeded assemblies. This is essential for understanding the seeded assembly of τ and for putting the prion concept on a more solid basis. The new structural work creates a rigorous baseline for future studies.

It is important to develop methods by which one can assemble recombinant τ into filaments like those formed in human brain. Only then will it be possible to study the mechanisms by which τ assembles into filaments of defined structure. Understanding these mechanisms is essential for the development of new therapeutic approaches. Knowledge of the near-atomic structures of τ filaments from human brain may lead to the development of improved diagnostic compounds.

## Summary

- τ filament folds differ between AD (Alzheimer fold), CTE (CTE fold), Pick's disease (Pick fold) and CBD (CBD fold), establishing the existence of molecular conformers of assembled τ. Differences are between some diseases, not between individuals with a given disease.
- The Alzheimer τ fold is also found in PART, indicating that this fold can form in the absence of Aβ deposits.
- Known τ filament structures from human brain differ from those of filaments assembled *in vitro*. It is now important to develop methods for assembling recombinant τ into filaments like those formed in human brain. Only then will it be possible to study mechanisms by which τ can assemble into filaments of defined structure.

## Open Access

Open access for this article was enabled by the participation of MRC Laboratory of Molecular Biology in an all-inclusive *Read & Publish* pilot with Portland Press and the Biochemical Society under a transformative agreement with JISC.

## Abbreviations

AD: Alzheimer's disease
AGD: argyrophilic grain disease
CBD: corticobasal degeneration
CTE: chronic traumatic encephalopathy
cryo-EM: electron cryo-microscopy
GGT: globular glial tauopathy
immuno-EM: immunoelectron microscopy
NPF: narrow Pick filament
PART: primary age-related tauopathy
PiD: Pick's disease
PET: positron emission tomography
PHF: paired helical filament
PSP: progressive supranuclear palsy
SF: straight filament
WPF: wide Pick filament
